# PD-L1 Influences Cell Spreading, Migration and Invasion in Head and Neck Cancer Cells

**DOI:** 10.3390/ijms21218089

**Published:** 2020-10-29

**Authors:** Jonas Eichberger, Daniela Schulz, Kristian Pscheidl, Mathias Fiedler, Torsten Eugen Reichert, Richard Josef Bauer, Tobias Ettl

**Affiliations:** 1 Department of Oral and Maxillofacial Surgery, University Hospital Regensburg, 9305 Regensburg, Germany; Jonas.Eichberger@stud.uni-regensburg.de (J.E.); daniela.schulz@ukr.de (D.S.); Kristian.Pscheidl@stud.uni-regensburg.de (K.P.); mathias.fiedler@web.de (M.F.); torsten.reichert@ukr.de (T.E.R.); tobias.ettl@ukr.de (T.E.); 2 Department of Oral and Maxillofacial Surgery and Center for Medical Biotechnology, University Hospital Regensburg, 9305 Regensburg, Germany

**Keywords:** PD-L1, head and neck squamous cell carcinoma, HNSCC, Rho-GTPase, migration, invasion

## Abstract

The programmed cell death protein-1 (PD-1)/programmed cell death ligand-1 (PD-L1) axis blockade has been implemented in advanced-stage tumor therapy for various entities, including head and neck squamous cell carcinoma (HNSCC). Despite a promising tumor response in a subgroup of HNSCC patients, the majority suffer from disease progression. PD-L1 is known to influence several intrinsic mechanisms in cancer cells, such as proliferation, apoptosis, migration and invasion. Here, we modulated PD-L1 expression in three HNSCC cell lines with differential intrinsic PD-L1 expression. In addition to an alteration in the epithelial-to-mesenchymal transition (EMT) marker expression, we observed PD-L1-dependent cell spreading, migration and invasion in a spheroid spreading assay on four different coatings (poly-L-lysine, collagen type I, fibronectin and Matrigel^®^) and a chemotactic transwell migration/invasion assay. Furthermore, the overexpression of PD-L1 led to increased gene expression and small interfering ribonucleic acid (siRNA) knockdown and decreased gene expression of Rho-GTPases and related proteins in a RT^2^ Profiler™ PCR Array. Rac1 and Rho-GTPase pulldown assays revealed a change in the activation state concordantly with PD-L1 expression. In summary, our results suggest a major role for PD-L1 in favoring cell motility, including cell spreading, migration and invasion. This is presumably caused by altered N-cadherin expression and changes in the activation states of small Rho-GTPases Rho and Rac1.

## 1. Introduction

Head and neck cancer is the seventh most common form of malignancies worldwide, accounting for more than 880,000 new cases and 453,000 fatalities in 2018, and is particularly increasing in low- to middle-income countries [1]. The most effective treatment is a multidisciplinary approach including surgery, radio- and chemotherapy and increasingly targeted therapies. While surgery or radiotherapy alone are the established methods for early stage I or II disease, multimodal therapy is necessary for patients with locally advanced stages of cancer [2]. Immunotherapy plays a major role in recurrent or metastatic head and neck cancer therapy. The programmed cell death protein-1 (PD-1)/programmed cell death ligand-1 (PD-L1) axis blockade has achieved revolutionary therapeutic success [3,4,5]. Meanwhile, anti-PD-1 drugs nivolumab and pembrolizumab are Food and Drug Administration (FDA)-approved to treat recurrent and metastatic head and neck squamous cell carcinoma (HNSCC). PD-1 is a 55 kDa type I transmembrane protein present on a variety of cells, including the membrane of thymocytes during selection as well as on mature B- and T-cells, antigen-presenting cells (APCs) and activated monocytes [6,7,8]. The expression of its two main ligands, PD-L1 and programmed cell death ligand 2 (PD-L2), can be constitutive or inducible. PD-L1 is found in lymphatic organs such as spleen and thymus. It is also expressed on a variety of different nonhematopoietic tissues, including cancer cells. The presence of PD-L2 is restricted to dendritic cells mainly in the thymic cortex [9]. The expression of PD-L1 is, for instance, induced by the activation of toll-like receptors and interferon (IFN)-γ-activated signaling pathways [10,11]. The interaction with PD-1 ultimately results in the suppression of T-cell response by the dephosphorylation of signaling cascades downstream to the T-cell receptor [12], which is physiologically desirable for central and peripheral tolerance under normal conditions. During cancer progression, tumor cells exploit the PD-1/PD-L1 signaling axis to evade the immune system. So far, PD-L1 expression has been found in various tumor entities, including breast, lung and colorectal cancer, glioblastoma, melanoma and head and neck cancers. In all these tumors, PD-L1 expression was strongly associated with poor prognosis [13,14,15,16,17,18]. Studies in patients with recurrent and/or metastatic head and neck squamous cell carcinoma showed a significantly better overall survival with the clinical use of antibodies against PD-1, such as nivolumab and pembrolizumab [19,20]. Part of this patient cohort still suffers from little improvement or even disease hyperprogression. The reason why patients respond differently to anti-PD-1 treatment is not clear yet. However, it could be due to an immune-system-independent mode of action of PD-L1. Several independent studies have recently suggested that PD-L1 may have intrinsic effects on cellular behavior [21,22,23]. Here, we present data demonstrating the role of PD-L1 in cell motility, including cell spreading, migration and invasion, thus creating the potential for lymph node and distant metastasis. Recent studies have shown a positive correlation between PD-L1 and epithelial-to-mesenchymal transition (EMT) expression in epithelial tumors, suggesting higher migrational and invasive capabilities [24,25,26,27]. Therefore, we investigated the possible role of PD-L1 in cell migration and invasion by studying the expression levels of epithelial and mesenchymal markers and conducting various functional assays with different types of HNSCC cell lines. We identified different genes involved in cell motility that could be possibly regulated by PD-L1 by performing RT^2^ Profiler™ PCR Array Human Cell Motility. Furthermore, triggered by the results of those arrays, we investigated the different expression levels and activation states of small GTPases of the Rho family, enzymes that are vital for the organization and stabilization of the cytoskeleton.

## 2. Results

Our previous studies revealed HNSCC cell lines with low (PCI 13), moderate (PCI 8) and high (PCI 52) PD-L1 expression. We demonstrated that PD-L1 expression is associated with radioresistance, proliferation and the expression of markers for the epithelial-to-mesenchymal transition (EMT) [28].

**Figure 1:**
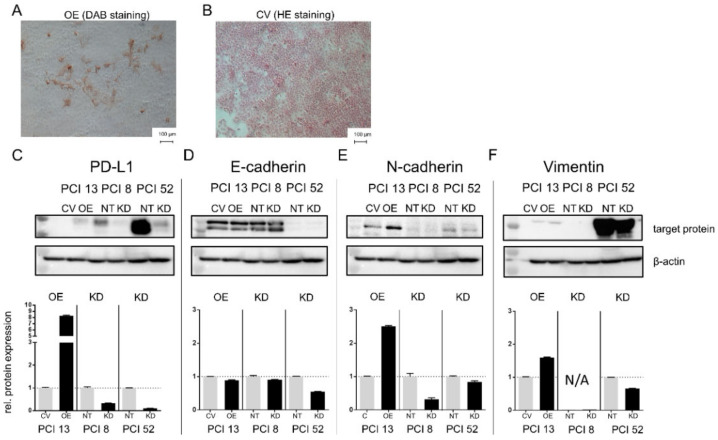
PD-L1 dependent expression of EMT markers. (**A**) Programmed cell death ligand-1 (PD-L1)-dependent expression of epithelial-to-mesenchymal transition (EMT) markers in 3D cultured cells may lead to morphological changes. PD-L1 overexpression in the head and neck squamous cell carcinoma (HNSCC) cell line PCI 13 with low intrinsic PD-L1 expression. Immunocytochemical staining reveals that PD-L1 overexpression resulted in cells with an elongated spindle-shaped morphology in contrast to cells transfected with the empty control vector cells (**B**). (**C**–**F**) Western blot analysis of PCI 13 after PD-L1 overexpression (OE) and PCI 8/PCI 52 after PD-L1 knockdown (KD). E-cadherin expression and expression of the mesenchymal markers N-cadherin and Vimentin are associated with PD-L1 expression. Lower lane: semiquantitative evaluation of Western blots for relative protein expression. Treated cells were normalized to their respective control. For Western blot analysis, 30 µg total protein lysate was used. β-actin served as a loading control. The results are expressed as means ± SD (standard deviation) for two experiments. CV = cells transfected with the empty control vector, OE = cells transfected with a vector containing the coding sequence for PD-L1, NT = cells transfected with nontargeting small interfering ribonucleic acid (siRNA) control, KD = knockdown cells transfected with siRNA against PD-L1.

Immunocytochemical staining of PD-L1-overexpressing HNSCC cells revealed an elongated, spindle-shaped morphology, which additionally indicates EMT. In contrast, cells transfected with a control vector that were subsequently stained by HE showed a characteristic consistent cobblestone morphology typical for epithelial cells (Figure 1).

Cells with a switch in the EMT program are often equipped with an increased migratory capacity and changes in integrin-dependent adhesion [29]. Therefore, we hypothesized that PD-L1 expression might be associated with migration and/or invasion changes, which also affects cytoskeletal protein expression. In the following experiments, PD-L1 expression was reduced via siRNA knockdown in intrinsically high and moderate PD-L1-expressing cell lines PCI 8 and PCI 52 and overexpressed via plasmid transfection in the low PD-L1-expressing cell line PCI 13.

Figure 1C indicates the efficiency of PD-L1 overexpression (OE) and knockdown (KD) in 3D cultured cells. HNSCC cell lines with low and moderate intrinsic PD-L1 expression (PCI 13 control vector (CV) and PCI 8 nontargeting (NT)) demonstrated a distinct E-cadherin expression, a marker for epithelial cell adhesion. In contrast, the cell line PCI 52 with a high intrinsic PD-L1 expression (NT) revealed only a minor E-cadherin expression. Here, either PD-L1 OE or KD did not markedly alter E-cadherin expression (Figure 1D). PCI 13 control cells (CV) revealed an intrinsic expression of N-cadherin and Vimentin with PD-L1 overexpression leading to the increased expression of both proteins (Figure 1E,F). PCI 8 control cells (NT) contained slight intrinsic N-cadherin levels, which were diminished by PD-L1 KD. PCI 52 showed a moderate intrinsic N-cadherin (NT) expression, which was only slightly influenced by PD-L1 KD (Figure 1E). There was a slight intrinsic Vimentin protein expression in PCI 13 control cells (NT) and strong expression in the PD-L1 high-expressing cell line PCI 52 (CV). Here, Vimentin expression was influenced by PD-L1 OE and KD (Figure 1F).

We hypothesized that PD-L1 expression might have an impact on cell spreading. Therefore, we investigated whether the modulation of PD-L1 expression in HNSCC cell lines with different PD-L1 intrinsic expression levels affects the capability of HNSCC cells’ spreading on surfaces coated with different extracellular matrix components (poly-L-lysine, collagen type 1, fibronectin and Matrigel^®^ (laminin-rich matrix)).

**Figure 2:**
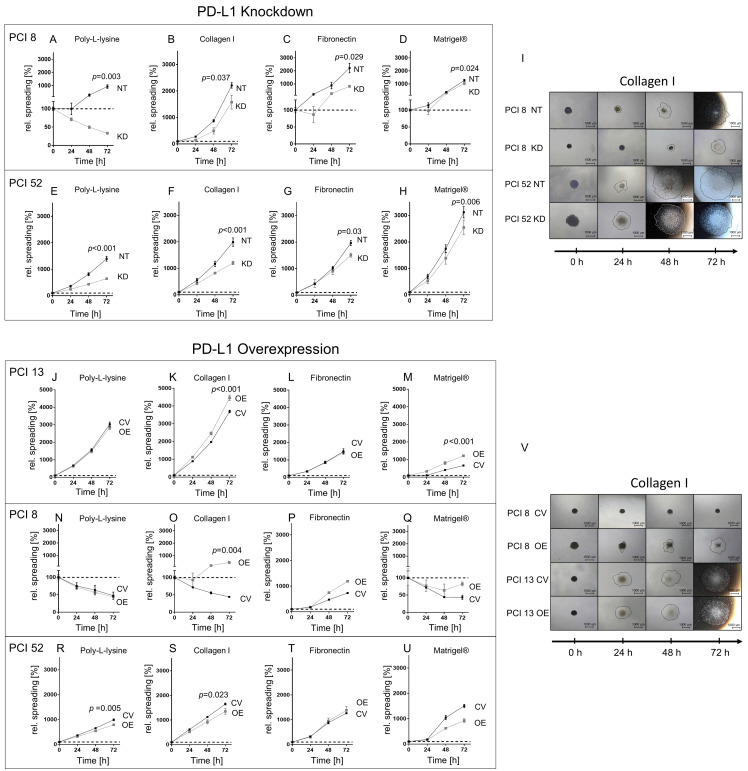
PD-L1-dependent cell spreading on various matrices. Spheroids from cell lines with low (PCI 13), moderate (PCI 8) and high PD-L1 expression (PCI 52) were seeded onto four differently coated surfaces: collagen type I, fibronectin and Matrigel^®^, a laminin-rich matrix. Poly-L-lysine served as control. Cell spreading was measured over a period of 72 h. The dotted line represents the initial area. (**A**–**H**) Spreading of PCI 8 and 52 spheroids after PD-L1 KD. (**I**) Exemplified images of cell spreading of NT and PD-L1 KD PCI 8 and PCI 52 spheroids on collagen type I. (**J**–**U**) Cell spreading of PD-L1 OE spheroids of PCI 13, PCI 8 and PCI 52. (**V**) Exemplified images of CV and PD-L1 OE PCI 8 and PCI 13 on collagen type I. Scale bar = 1000 µM, black line depicts spreading area measured and calculated with the ImageJ software. The results are expressed as means ± SD, *n* = 4; endpoints after 72 h were statistically compared by unpaired Student’s *t*-test.

Figure 2 shows the spreading of HNSCC spheroids with low (PCI 13), moderate (PCI 8) and high (PCI 52) intrinsic PD-L1 expression after siRNA knockdown or overexpression on coatings with different matrix components within 72 h. Poly-L-lysine coating served as a control coating for nonspecific integrin-independent adhesion.

PD-L1 KD in PCI 8 spheroids with an intrinsically moderate PD-L1 expression led to cell death on the poly-L-lysine coating resulting in a decrease in the spread of the area. The addition of matrix components led to cell spreading. However, spreading on each of the coatings was significantly reduced in KD cells compared to NT controls (Figure 2).

Compared to poly-L-lysine, PD-L1 KD in intrinsically high PD-L1-expressing PCI 52 spheroids did not show a significant change in their extent of spreading on different matrix components compared to the poly-L-lysine coating. However, when comparing KD cells with NT controls, the spreading of KD cells was significantly reduced in each case.

Additionally, we investigated if PD-L1 OE also affected cell spreading in high and moderate PD-L1-expressing cells PCI 52 and PCI 8. PD-L1 OE in high-expressing PCI 52 spheroids did not impact cell spreading on any of the coatings. Interestingly, PD-L1 OE led to increased cell spreading in moderately-expressing PD-L1 spheroids (PCI 8) on the collagen type I surface coating (Figure 2P,V).

Furthermore, PD-L1 OE in low PD-L1-expressing PCI 13 spheroids demonstrated a significant increase in spreading on coatings with collagen type I (1.2-fold) and Matrigel^®^ (1.8-fold) (Figure 2K,M,V).

**Figure 3:**
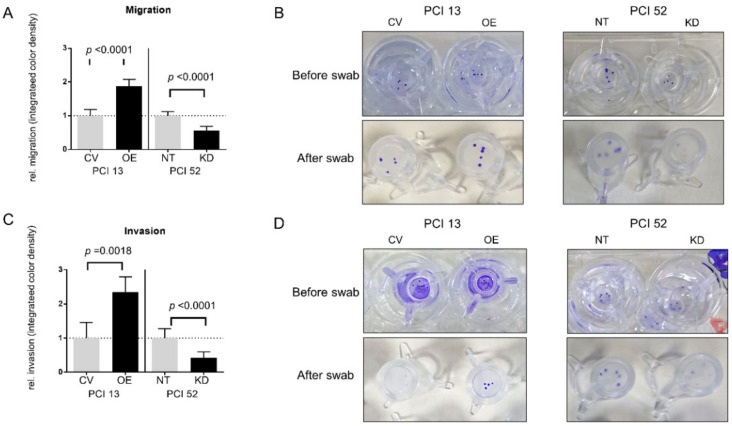
Influence of PD-L1 on migration and invasion. To evaluate the role of PD-L1 in the migration and invasion of HNSCC cell lines along a chemotactic nutrient gradient, a transwell Boyden chamber assay was conducted. (**A**,**B**) Chemotactic migration of PCI 13 and PCI 52 spheroids with PD-L1 siRNA knockdown (KD) or overexpression (OE). PD-L1 overexpression in PCI 13 (OE) showed a significantly increased transwell migration of spheroids compared to control cells (CV). In contrast, there was a markedly reduced migration after siRNA knockdown (KD) in PCI 52 cells compared to control cells (NT). Accordingly, PD-L1 overexpression led to a significantly increased chemotactic invasion of PCI 13 through a thin layer of Matrigel^®^ and siRNA knockdown in PCI 52 to a marked reduction (**C**,**D**). (**B**,**D**) Photographic documentation of transwell migration assays before and after swabbing nonmigrated/noninvaded cells. The results are expressed as means ± SD, *n* = 4; unpaired Student’s *t*-test.

As we observed a PD-L1-dependent difference in the spreading of low-, moderate- and high-expressing HNSCC spheroids on different coatings, we hypothesized that cells would also show PD-L1-dependent migration and invasion along a chemotactic gradient. For this purpose, we observed the behavior of spheroids in Boyden chamber assays. Spheroids of cell lines PCI 13 with an intrinsically low and PCI 52 with an intrinsically high PD-L1 expression were used in the transwell assays. After the modulation of PD-L1 expression, differences in chemotactic migration and invasion were observed in all spheroids compared to their respective controls, which were nontarget (NT) for siRNA knockdown and empty control vector (CV) for overexpression experiments (Figure 3). Overall, after PD-L1 OE in intrinsically low PD-L1-expressing spheroids (PCI 13), we noticed a significant increase in migration and invasion along the chemotactic gradient. Migration after OE increased by about 1.9-fold. In addition, there was an average 2.4-fold increase in invasion after PD-L1 OE. On the contrary, after siRNA KD, there was a significant 1.8-fold reduction in migration and about a 2.4-fold reduction in invasion compared to control cells in high basal PD-L1-expressing cells (PCI 52).

**Figure 4:**
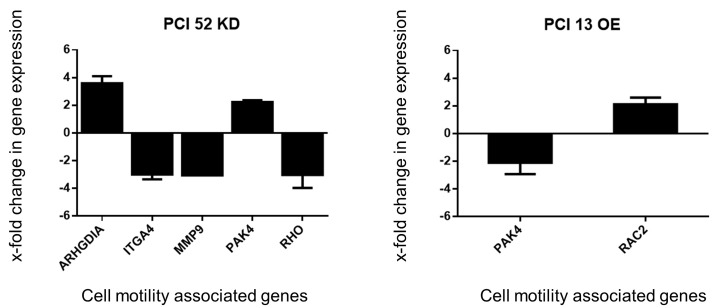
PD-L1-dependent gene expression change. Analysis of PD-L1-dependent expression of cell motility associated genes. Extract from the RT^2^ Profiler™ PCR Array Human Cell Motility for genes relevant to cytoskeletal organization. PD-L1 modulation affects various genes associated with Rho-GTPase regulation. Only genes with more than a 2.0-fold change in gene expression were considered to be relevant for evaluation. KD = siRNA knockdown of PD-L1. OE = plasmid overexpression of PD-L1. For qPCR, 25 ng of cDNA was used. The results were expressed as means ± SD; *n* = 4.

Based on the previous results, we assumed that PD-L1 might influence the gene expression of proteins that are part of the cytoskeletal structure and its organization. To obtain an insight into whether there is a PD-L1-dependent expression of cytoskeletal-associated genes, a corresponding RT^2^ Profiler™ PCR Array Human Cell Motility array was performed. The intrinsically low and high PD-L1-expressing PCI 13 and PCI 52 were used exemplarily. In PCI 13 spheroids, PD-L1 was transiently overexpressed, and in PCI52 spheroids, a transient siRNA knockdown was performed. After PD-L1 modulation, the expression of several genes related to cell motility and cytoskeletal organization was affected such that they differed by at least two-fold from the expression in NT or CV controls: RAC2, ARGHDIA, PAK4, ITGA4, MMP9 and RHO (Figure 4).

PD-L1 OE in PCI 13 showed an upregulation of RAC2 gene expression (2.12-fold), which encodes for the corresponding protein Rac2, a member of the small GTPase family. PAK4 gene expression, a serine/threonine kinase known as an effector protein of the small Rho-GTPases, was downregulated (2.58-fold) compared to cells transfected with the empty control vector (Figure 4, right chart). PD-L1 KD in PCI 52 revealed an upregulation of ARGHDIA (3.59-fold), a modulator of Rho-GTPases and an upregulation of PAK4 (2.24-fold) gene expression. Additionally, the data showed downregulation of the ITGA4 (2.99-fold) gene, MMP9 (3.06-fold) and RHO (3.03-fold) gene expression (Figure 4, left chart).

**Figure 5:**
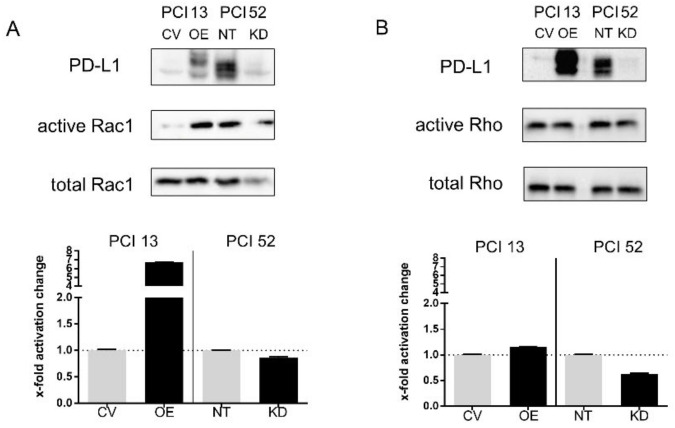
PD-L1-dependent modulation of Rho-GTPase activation states. (**A**) PD-L1-dependent activation of small GTPases Rac1 and Rho. Western blot analysis of cell lysates from Rho-GTPase pulldown assays of PD-L1-overexpressing PCI 13 and PCI 52 with siRNA knockdown of PD-L1. Transfection with nontargeting siRNA was used as control (NT). **A** Representative WB analysis with the precipitate from Rho-GTPase pulldown assays. (**B**) Semiquantitative analysis revealed a decreased activation state of Rho in PCI 52 and an increased activation state of Rac1 in PCI 13 when compared to control. The results are expressed as means ± SD; *n* = 2.

Encouraged by these results, we decided to look further into the activation state of the Rho family of small GTPases by means of a Rho/Rac1/Cdc42 pulldown assay. A previous Western blot analysis to determine the amount of lysate required for the pulldown did not detect any Cdc42 protein. Therefore, Cdc42 was neglected for further analysis. However, Rac1 and Rho displayed altered activation states depending on PD-L1 modulation in PCI 13 and PCI 52 with intrinsically low and high PD-L1 expression, respectively. The overexpression of PD-L1 in PCI 13 (OE) led to a > 6-fold increase of Rac1 activity compared to control cells (CV) (Figure 5A). In contrast, there was almost no change in Rho activation (Figure 5B). Conversely, siRNA knockdown of PD-L1 in PCI 52 revealed a marked decrease of Rho activation but no change in Rac1 activation. This is consistent with our results from the qPCR arrays, where RHO gene expression was decreased in PCI 52 knockdown and RAC2 expression was increased in PD-L1-overexpressing PCI 13.

## 3. Discussion

The role of PD-L1 for patient survival and disease-related complications such as locoregional recurrence remains controversial. On the one hand, PD-1/PD-L1 interaction favors tumor progression by allowing malignant cells to evade immune cells. Activation of this pathway leads to the silencing of tumor-infiltrating lymphocytes, especially CD8^+^ T-cells [30]. Beyond this, PD-L1 influences various intrinsic mechanisms in the tumor cell. Abundant evidence in the literature implies an association between PD-L1 and EMT [25,26,27,31,32,33] and their capability to influence cell migration and invasion. Moreover, the development of PD-L1-positive metastases suggests higher mortality [34]. A meta-analysis of studies conducted on gastric cancer patients revealed a relationship between PD-L1 expression levels and poor prognosis. In addition, a subgroup analysis revealed that patients with a PD-L1-positive tumor were prone to lymph node metastasis and deeper invasion of tumor-surrounding healthy tissue [35]. Furthermore, Müller et al. discovered that high PD-L1 expression in HNSCC contributed to an aggressive cancer phenotype, diminishing overall survival in their cohort [36]. However, some studies indicate the opposite. Through immunohistochemistry of 95 tissue samples of HNSCC, Chen et al. demonstrated increased overall survival and a lower chance of recurrence in PD-L1-positive samples [37]. Additionally, PD-L1 mRNA and protein positivity were significantly associated with longer overall survival [38]. In the current work, we observed PD-L1-dependent spreading on differently coated surfaces. Moreover, we found different migration and invasion potentials of HNSCC spheroids. These results were accompanied by PD-L1-dependent EMT marker expressions like N-cadherin and Vimentin. Our experiments are in line with the literature as there has been evidence that PD-L1 might influence EMT in a variety of tumors and favors locoregional growth and distant metastasis in clinical patients. A decrease in E-cadherin and an increase in Vimentin was found by Ock et al. to have a poor prognosis in a cohort of patients with high PD-L1 expression. as well as a simultaneous expression of EMT markers [27]. In renal cancer cell lines, lentiviral PD-L1 knockdown led to low expression levels of Vimentin and higher levels of E-cadherin. In accordance with our data, they observed a change in cell morphology where cells would regain their epithelial cobblestone-like shape. They showed that the overexpression of PD-L1 resulted in the opposite effect of the knockdown, where Vimentin expression levels rose while E-cadherin was decreased. Additionally, they reported an increase in specific pluripotency regulation markers after PD-L1 overexpression, which led to an increased spheroid formation in PD-L1-overexpressing cells, suggesting EMT stimulation by PD-L1 [31]. Other groups found significantly increased Ras activity when PD-L1 was present, suggesting EMT activation via the Ras-MEK/ERK-EMT pathway, which led to the contribution of the mesenchymal phenotype. A transfer to an in vivo model also showed more aggressive tumor growth and extended invasion of surrounding healthy tissue in rats [25,32,33].

We demonstrate that PD-L1 plays a role in HNSCC cell migration and invasion. Our results are concordant to a variety of clinical and experimental data in the literature. Not only would migration and invasion result in distant metastasis, but it would also favor the invasion of adjacent lymph nodes as well. Two independent studies reported a positive correlation between PD-L1 expression and lymph node metastasis in HNSCC. Schneider et al. investigated 129 tissue samples of oral cavity cancer patients and 77 corresponding lymph node metastases. PD-L1 positivity in the primary tumor significantly correlated with the occurrence of invaded lymph nodes. Patients with a PD-L1-expressing tumor also showed significantly lower disease-free and overall survival [24]. By analyzing a sample of 80 predominantly HPV-negative tissues, Straub et al. found 72% of primary tumor and corresponding lymph node metastases to be PD-L1 positive. In this study, PD-L1 expression was additionally associated with a higher probability of tumor-related death and tumor recurrence [39]. Recent data also show an association of PD-L1 regulation together with EMT signaling. Xu et al. were able to demonstrate a TGF-β1-dependent PD-L1 expression in a gastric cancer cell line. They concluded cytokine-mediated EMT promoted PD-L1 expression and triggered invasion and migration. [40]. Moreover, Yan et al. showed an increased migration in breast cancer cell lines when overexpressing PD-L1 by means of IFN-γ treatment. They noticed a decrease in E-cadherin and an increased N-cadherin and Vimentin expression compared to the controls. [41]. Fei et al. demonstrated a PD-L1-dependent change in cell migration and invasion in nasopharyngeal cancer cells. Interestingly, they observed an upregulation of the PI3K/Akt-pathway [42]. Qiu et al. reported increased migration in scratch wound healing assays of PD-L1-overexpressing glioblastoma multiforme cell lines, whereas PD-L1 knockdown cells showed the opposite effect [43]. We observed a marked increase in cell spreading on collagen type I and Matrigel^®^ after the overexpression of PD-L1, particularly in PCI 13 and PCI 8, which only had very low or moderate intrinsic PD-L1 basal expressions. In contrast, the overexpression of PD-L1 in PCI 52 with an abundant intrinsic PD-L1 expression led to a decrease in cell spreading on collagen type I and Matrigel^®^. This might be due to the overload of PD-L1 expression (intrinsic plus overexpression), where cells have to prevent the aggregation of excessive proteins, which is toxic for cells at a certain concentration [44]. On the other hand, the siRNA knockdown of PD-L1 led to the decreased spreading of all HNSCC spheroids independent of surface coatings. One noticeable aspect of these experiments was the spreading behavior of PCI 8 on poly-L-lysine. After siRNA knockdown, these cells almost failed to spread at all. When the matrix was available, they exhibited spreading behavior similar to the other cell lines with PD-L1 knockdown. Knockdown in PCI 8 led to an almost complete loss of N-cadherin, whereas E-cadherin levels remained unchanged. This promotes a high grade of cell–cell adhesion, while poly-L-lysine has shown to inhibit cell mobility [45]. PCI 8 has small amounts of intrinsic N-cadherin and no Vimentin. Hence, the lack of N-cadherin expression after PD-L1 knockdown could be one reason for the very low spreading of cells without a matrix [46,47].

Interestingly, our qPCR analysis for cell-motility-related genes revealed PD-L1-dependent gene regulation associated with Rho-GTPases and genes coding for effector proteins of the Rho family of small GTPases. Rho and Rac1 are vital for the organization of the actin cytoskeleton. Physiologically, Rho regulates the formation of stress fibers and Rac GTPases are responsible for the formation of lamellipodia and membrane ruffling [48]. In malignancies, Rho and Rac1 are suspected to influence EMT by regulating the degradation or stabilization of cadherins, whose protein expression levels we saw modified. In addition, they are able to regulate the expression level of matrix metalloproteinases (MMPs) [49], for which we observed a more than three-fold reduction in the gene expression of MMP9. ARGHDIA activation leads to the expression of Rho-GDP-dissociation inhibitors, which prevent the dissociation of GDP from Rho-GTPases [50]. PAK4 is a serine/threonine kinase that also acts as an effector protein. There has been evidence that PAK4 drives prostate and colon cancer cells to metastasize [51,52]. The PD-L1 overexpression of PCI 13 showed a more than six-fold increase in Rac1 activity, which could indicate a cause for enhanced cell motility.

Although the prognostic role of PD-L1 is not yet clear, most studies show an association between PD-L1 and the occurrence of local and distant metastases in in vivo and in vitro data. Our results provide further insight into the different mechanisms of the association between PD-L1, EMT, cell migration and invasion in HNSCC. Moreover, we suggest PD-L1 has a possible role as a regulator of the activity of a subgroup of Rho-GTPases, thus influencing the organization of the cytoskeleton, causing differential behavior during spread, migration and invasion.

## 4. Materials and Methods

### 4.1. Cell Lines and Culture Conditions

Human HNSCC cell lines were kindly provided by Prof. Dr. Theresa. L. Whiteside (University of Pittsburgh Cancer Institute (PCI), Pittsburgh, PA, USA). After a detailed characterization of the HNSCC cell lines, we found cell lines with low (PCI 13), moderate (PCI 8) and high (PCI 52) PD-L1 expression. The cells were derived from the primary tumors of different anatomical localizations: PCI 13—retromolar triangle, PCI 8—Piriform sinus and PCI 52—Plica aryepiglottica [49,53]. Cells were maintained in DMEM (PanBiotech, Aidenbach, Germany) supplemented with 10% fetal calf serum (FCS, Life Technologies, Carlsbad, CA, USA), 1% L-glutamine (Sigma-Aldrich, Munich Germany) and 1% penicillin/streptomycin (Sigma-Aldrich) at 37 °C in a 5% CO_2_ humidified atmosphere. The medium was changed every two to three days. Cells were passaged prior to reaching confluence. They were detached by incubation with 0.05% trypsin-EDTA solution (Sigma-Aldrich) for 5 to 10 min at 37 °C and transferred into a new cell culture flask in the appropriate cell count.

### 4.2. 3D Cell Culture

To investigate cell properties in 3D cell culture, 3D spheroids were cultured via the hanging drop method, first described by Harrison et al. [54]. Therefore, adherent HNSCC cells were cultivated up to 70% confluence. Cells were detached with StemPro^®^ Accutase (Life Technologies). After cell counting, they were centrifuged (Hettich centrifuge, Tuttlingen, Germany, 1160 rpm, 220× *g*, 5 min) and resuspended with growth medium supplemented with 10% methylcellulose of a 1.2% stock solution, resulting in a final concentration of 0.12% methylcellulose. (Sigma-Aldrich). Drops with a volume of 20 µL containing 20,000 cells were then seeded on the lower side of the lid of a Petri dish. After seeding, the lid was inverted. The bottom of the Petri dish was filled with 5 mL PBS (Life Technologies) to minimize the evaporation of the medium during incubation. Afterwards, the spheroids were incubated for 24 h at 37 °C in a 5% CO_2_ humidified atmosphere. After 24 h, spheroids were used in the respective assays.

### 4.3. Transient siRNA Knockdown of PD-L1

To deplete PD-L1 expression (KD), cells were transiently transfected with small interfering RNA (siRNA) targeted against the human PD-L1 gene CD274. Dharmafect-1 (Thermo Scientific, Waltham, MA, USA) was used as a transfection reagent according to the manufacturer’s instructions. Transfection was performed in 6-well plates (Corning, NY, USA). For reverse transfection, 1–4 × 10^5^ freshly passaged cells were added to preplated transfection complexes. Transfection with 25 nM siRNA was performed in DMEM growth medium with 1% L-glutamine and 10% FCS (without antibiotics) for 72 h. The siGENOME Human CD274 siRNASMARTpool (Horizon, Cambridge, UK) consisted of four siRNA target sequences. The ON-TARGETplus Nontargeting Pool (Horizon) was used as a control. SiRNA sequences are listed in Table 1.

Three days after siRNA transfection, cells were used for experiments. The expression of PD-L1 in HNSCC cell lines was determined at the beginning and end of each experiment using Western blot analysis. At the beginning of the experiment, siRNA-transfected cells showed a PD-L1 reduction of approximately 80%. Transient siRNA knockdown was efficient for at least the duration of the observation.

### 4.4. Transient Plasmid Overexpression of PD-L1

For PD-L1 overexpression (OE), cells were transiently transfected with a plasmid carrying a gene sequence coding for the human PD-L1 gene CD274. FuGene HD transfection reagent (Promega, Fitchburg, WI, USA) was used according to the manufacturer’s instructions. The expression vector PD-L1 pcDNA 3.1 (+) was designed and cloned by Invitrogen Thermo Scientific in *Escherichia coli* K12 DH10B™ T1R. The sequence length of PD-L1 was 891 bp. The length of the whole plasmid was 6248 bp. CV pcDNA 3.1 (+) (control vector (CV)) without an insert (Thermo Scientific) was used as a control. Twenty-four hours prior to transfection, 4 × 10^5^ cells were seeded in 6-well plates (Corning, Corning, NY, USA). The culture medium during transfection was DMEM with 1% L-Glutamine (without FCS and antibiotics). Reaction complexes were formed from the combination of FuGene HD transfection reagent and plasmids diluted in DMEM in a ratio of 5:2 (5 μL FuGene HD per 2 μg DNA). Adherent cells were transfected for 16–24 h. Afterwards, cells were used for experiments. The expression of PD-L1 in HNSCC cell lines was determined at the beginning and the end of each experiment using Western blot analysis. At the beginning of the experiment plasmid, transfected cells showed an approximately 4-fold higher PD-L1 expression than cells transfected with CV. Transient plasmid overexpression was efficient for at least the duration of the observation.

### 4.5. Immunocytological DAB Staining of PD-L1

For the immunocytological DAB staining of PD-L1, cells were seeded in 4-chamber slides (BD Biosciences, Falcon, MA, USA). At the time of the analysis, cells were fixed in 2% paraformaldehyde (min. 37%) (Merck, Darmstadt, Germany) for 10 min. Prior to staining, endogenous peroxidase activity was blocked by incubating cells with 10% H_2_O_2_ (hydrogen peroxide solution) (Sigma-Aldrich, Munich, Germany) and 10% MeOH (Carl Roth, Karlsruhe, Germany) for 10 min at room temperature. Cells were incubated with 5% goat serum (Sigma-Aldrich) in PBS (Life Technologies) blocking solution for 1 h in order to prevent nonspecific antibody binding. The anti-PD-L1 primary antibody (rabbit mAb, clone E1L3N, #13684, Cell Signaling, Boston, MA, USA) was diluted to 3 µg/mL in 3% BSA in PBS (Life Technologies) and incubated overnight at 4 °C. A polyclonal rabbit IgG antibody (I5006, Sigma-Aldrich, Munich, Germany) was used as the isotype control at the same concentration. For signal detection, slides were incubated with EnVision™+ Dual Link System-HRP (DAKO, Agilent, Santa Clara, CA, USA) for 10 min. Visualization of the immunoreactivity was performed with 3,3′-diaminobenzidine (DAB) tablets (Sigma-Aldrich). Slides were incubated for approximately 10 min until DAB staining was sufficiently visible. For counterstaining, sections were mounted in VECTASHIELD^®^ Antifade Mounting Medium containing the fluorescent DNA binding dye DAPI (Vector Laboratories, Burlingame, CA, USA). The slides were coverslipped and digitally photographed in brightfield illumination at 4-fold magnification under a microscope (Nikon Eclipse TE2000-U, Nikon, Minato, Japan).

### 4.6. HE Staining of HNSCC Cells

For visualization of adherent cells, HE staining was performed. At the time of the analysis, cells were fixed in 2% paraformaldehyde (min. 37%) (Merck, Germany) for 10 min. This was followed by two washing steps with H_2_O for 2 min each. For the nucleus staining, the slides were incubated in a hemalum solution (Carl Roth) for 5 min. For blue staining, the slides were placed in running tap water for 10 min. Counterstaining in a 1% aqueous eosin solution (Carl Roth) lasted 1 min. Excess staining solution was removed in H_2_O in a short washing step. Before mounting, the stained cells were dehydrated with ascending alcohol series: 70% ethanol for 10 s (s), 96% ethanol for another 10 s and finally twice with 100% ethanol for 2 min each. Afterwards, the slides were incubated with xylene for 1–2 min and then covered with the xylene-containing mounting medium Vitro-Club^®^ (R. Langenbrinck GmbH, Emmendingen, Germany). The slides were coverslipped and digitally photographed in brightfield illumination at a 4-fold magnification under a microscope (Nikon Eclipse TE2000-U, Nikon, Minato, Japan).

### 4.7. Spheroid Spreading Assay

Three-dimensional (3D) spheroids were cultured for 24 h. To assess cell spreading, HNSCC cells were seeded onto 24- and 96-well plates (Cellstar^®^, Greiner Bio-One GmbH, Kremsmünster, Austria), respectively, coated with four different matrices: poly-L-lysine, which served as a control for integrin-independent attachment, collagen type I, fibronectin and Matrigel^®^, a laminin-rich matrix (Corning Inc., NY, USA). For the Matrigel^®^ coating, 50 μL of a 9.3 mg/mL Matrigel^®^ stock solution was used per 24-well in an ice-cooled condition. After coating, the Matrigel^®^ was allowed to dry for 30 min at 37 °C in a 5% CO_2_ humidified atmosphere. The wells were filled with 500 μL (24-well) or 100 µL (96-well) growth medium. Images were taken under a microscope in 4-fold and 2-fold magnifications. They were taken immediately after seeding (0 h) as well as after 24 h, 48 h and 72 h of incubation. The diameter of the spheroid spreading area was measured with the ImageJ 1.52a software. Afterwards, the change in the size of the area covered by cells was calculated. Therefore, the ratio of the area after 72 h to the covered area right after seeding (0 h) was calculated and referred to as relative (rel.) spreading. Statistical analysis was done using the GraphPad Prism 6 software (GraphPad Software, San Diego, CA, USA).

### 4.8. Chemotactic Migration and Invasion Assay

In order to determine the influence of PD-L1 expression on chemotactic cell motility, we performed chemotactic Boyden chamber migration and invasion assays. These assays consist of two chambers: an upper chamber and a lower chamber. Five spheroids were seeded in a permeable insert (upper chamber) with an average pore size of 8 μm (ThinCert™ cell culture inserts for 24-well plates, (Greiner Bio-One GmbH, Kremsmünster, Austria), filled with 100 μL DMEM with 2% fetal calf serum, 1% L-Glutamine and 1% penicillin/streptomycin and hung in 24-well plates. The bottom of the well was filled with 500 μL DMEM with 10% fetal calf serum, 1% L-Glutamine and 1% penicillin/streptomycin to create a nutrition gradient. For invasion experiments, the ThinCerts were additionally coated with 50 µL of Matrigel^®^ with a concentration of 3 mg/mL (Corning Inc.) as previously described. For migration experiments, the inserts remained uncoated. Spheroids were put in a 5% CO_2_ humidified atmosphere for 48 h at 37 °C. Spheroids left on the top side of the upper chamber were removed with a cotton swab. Cells that migrated/invaded through the pores to the bottom side of the upper chamber were fixated with 1% glutaraldehyde (Merck, Germany) for 30 min at room temperature. The cells were dyed with a 0.02% solution of crystal violet (Sigma-Aldrich) for 15 min at room temperature. Excess dye was removed with tap water. For the quantification of migration/invasion, the stained cells were photographed before and after swabbing. For analysis, images were converted to black and white and inverted using and the integrated color density was calculated using Adobe. The integrated color density comprises the size of the spheroids and the color density.

### 4.9. Protein Isolation

Adherent cells were washed with PBS, scraped in radioimmunoprecipitation assay buffer (RIPA) (Sigma-Aldrich) with protease inhibitors (complete mini Protease Inhibitor Cocktail), (Roche, Mannheim, Germany). Three-dimensional (3D) cultured cells were collected with a pipette. Collected spheroids were washed with PBS (Life Technologies) before the RIPA buffer (Sigma-Aldrich) was added. To improve lysis, the harvested adherent cells and spheroids were sonicated on ice for 20 s. Cell pellets and supernatants were separated by centrifugation for 10 min at 14,000× *g* at 4 °C. The supernatant was stored at −20 °C for further analysis.

### 4.10. Western Blot Analysis

For quantitative protein analysis, 30 μg of total protein was used for Western blotting. Protein concentration was assessed by using a bicinchoninic acid (BCA) assay (Merck, Germany). Proteins were denatured at 70 °C for 10 min in Laemmli sample buffer (Bio-Rad, Hercules, CA, USA) containing 1% β-mercaptoethanol (Merck, Germany). Samples were separated by SDS-PAGE using a 10% resolving gel and transferred onto a PVDF membrane (Roche). The membrane was blocked with 5% skimmed milk (Carl Roth) in a TBS buffer containing 0.1% Tween 20 (Sigma-Aldrich) for 1 h at room temperature. This step was followed by incubation with a specific primary antibody at 4 °C overnight. Primary antibodies used for Western blot analysis were anti-PD-L1 (rabbit mAb, clone E1L3N, #13684, CST), anti-Rac1 (mouse mAb #240106, Cell Biolabs, Inc., San Diego, CA, USA) and anti-Cdc42 (mouse mAb #240201, Cell Biolabs, Inc, CA, USA). After removing the unbound primary antibody, the membrane was incubated with a secondary antibody conjugated with horseradish peroxidase (HRP). The secondary antibodies used for signal detection were goat antirabbit (#32460, Thermo Scientific) and goat antimouse stabilized peroxidase-conjugated (#32430, Thermo Scientific). Roti Lumin (Carl Roth) or SuperSignal West Femto Maximum Sensitivity Substrate (Thermo Scientific) was used as a substrate. Colorimetric and chemiluminescent pictures were processed with the high-resolution, high-sensitivity ChemiDoc XRS+ Imaging System (Bio-Rad). Equal loading of proteins was verified with a specific antibody against β-actin (mouse antirabbit polyAb ab8227 Abcam, Cambridge, UK). The housekeeping protein was incubated on the same membrane after membrane stripping using ReBlot Plus Strong Antibody Stripping Solution (Merck, Germany). Normalization and quantification were performed with the Image Lab software 5.2.1 (Bio-Rad).

### 4.11. RT^2^ Profiler™ PCR Array

In order to detect cell motility associated genes that correlate with PD-L1 expression, RT^2^ Profiler™ PCR Array was performed (Qiagen, Hilden, Germany). Therefore, total cellular RNA was isolated from cells using the RNeasy mini kit (Qiagen) according to the manufacturer’s instructions. To prevent contamination with genomic DNA, additional digestion with Rnase-free Dnase (Qiagen) was performed. Reverse transcription of 3 μg RNA to complementary DNA (cDNA) was performed using the transcriptor First Strand cDNA Synthesis Kit (Roche) according to the manufacturer’s protocol. cDNA obtained from transfected cell lines was mixed with RT^2^ Real-Time SYBR Green/ROX PCR master mix (Qiagen). Twenty-five microliters of the solution containing 25 ng of cDNA was then placed into each well of the 96-well array plate, comprising 84 migration-relevant genes and 12 controls. The amplification run consisted of one segment of 95 °C for 10 min, followed by 40 cycles of denaturation at 95 °C for 15 s and primer hybridization and elongation at 60 °C for 1 min, before it was concluded with a final segment of 95 °C for 1 min, 65 °C for 30 s and 95 °C for 1 min. A dissociation curve served as the quality control. Analysis was done online using a template provided from the Qiagen platform (https://dataanalysis2.qiagen.com/pcr). Samples were analyzed with the ΔΔct method. To determine fold change, the ratio of the gene expression in the treated cells is divided by the gene expression of the control.

### 4.12. Rho/Rac1/Cdc42 Activation Assay

To investigate the activation status of cell-motility-associated proteins depending on PD-L1 expression, a Rho/Rac1/Cdc42 activation assay was performed (Cytoskeleton, Inc., Denver, CO, USA) according to the manufacturer’s instructions. Transfected cells were cultivated in a T75 cell culture flask (Corning, NY, USA) of up to 80% confluence. Prior to the pulldown assay, cells were stimulated with a Rac1/Cdc42 Activator (Rac/Cdc42 Activator II, Cytoskeleton, Inc) according to the manufacturer’s instructions. For the analysis of activated Rho, stimulation of cells was not required. After stimulation, cells were washed with ice-cold PBS (Life Technologies) and an assay lysis buffer was added. Cells were scraped and then centrifuged (10 min, 14,000× *g* at 4 °C, Sigma 3K30). The supernatant was collected and kept on ice for immediate use. The cell lysate was aliquoted to 0.5–1 mL. The volume was adjusted with an assay lysis buffer before the PAK PBD agarose beads were added to the mixture. The mixture was incubated for 1 h at 4 °C, followed by centrifugation (10 s, 14,000× *g*); the supernatant was discarded. Beads were washed with assay lysis buffer and resuspended in SDS-PAGE buffer. Pulled-down proteins were boiled for 5 min before short centrifugation (10 s, 14,000× *g*). Protein analysis was performed via Western blotting. GTPγS or GDP mixed with the samples served as positive or negative loading controls. The proportion of activated Rho and Rac1 was analyzed using the Image Lab software 5.2.1 (Bio-Rad).

### 4.13. Statistical Analysis

For spheroid spreading assay, the significance of the difference in the spread was calculated between treated and control cell lines by Area at T = 72 h (mm^2^)/Area T = 0 h (mm^2^) = x-fold increase. The endpoints at 72 h were analyzed via Student’s *t*-test in the GraphPad Prism software (GraphPad Software, San Diego, CA, USA).

## Figures and Tables

**Table 1:** CD274 siRNA SMARTpool target sequences.

siRNA Target Sequences	Nontargeting Pool
AGACCUGGCUGCACUAAUU (D-015836-03)	UGGUUUACAUGUCGACUAA
UGAAAGGACUCACUUGGUA (D-015836-01)	UGGUUUACAUGUUGUGUGA
CAUAGUAGCUACAGACAGA (D-015836-02)	UGGUUUACAUGUUUUCUGA
GGACCUAUAUGUGGUAGAG (D-015836-04)	UGGUUUACAUGUUUUCCUA

## Abbreviations

PD-L1	Programmed cell death ligand-1
PD-1	Programmed cell death protein-1
HNSCC	Head and neck squamous cell carcinoma
siRNA	Small interfering ribonucleic acid
EMT	Epithelial–mesenchymal transition
FDA	Food and Drug Administration
PD-L2	Programmed cell death ligand 2
IFN	Interferon
APC	Antigen-presenting cell
PCI	Pittsburgh Cancer Institute
NT	Nontargeting
KD	Knockdown
CV	Control vector
OE	Overexpression
RAC2	Gene encoding for the small Rho-GTPase Rac2
ARGHDIA	Gene encoding for Rho-GDP-dissociation inhibitor 1
PAK4	Gene encoding for serine/threonine-protein kinase PAK 4
ITGA4	Gene encoding for integrin alpha 4
MMP9	Gene encoding for matrix-metalloproteinase 9
RHO	Gene encoding for the small Rho-GTPase Rho
qPCR	Quantitative polymerase chain reaction
DMEM	Dulbecco’s modified Eagle’s medium
FCS	Fetal calf serum
EDTA	Ethylenediaminetetraacetic acid
DAB	3,3′-Diaminobenzidine
PBS	Phosphate buffered saline
PVDF	Polyvinylidene fluoride
SDS-PAGE	Sodium dodecyl sulfate polyacrylamide gel electrophoresis
γ	Gamma
SD	Standard deviation
